# Dimethyl Fumarate (DMF) Alleviated Post-Operative (PO) Pain through the *N*-Methyl-d-Aspartate (NMDA) Receptors

**DOI:** 10.3390/antiox11091774

**Published:** 2022-09-08

**Authors:** Giovanna Casili, Marika Lanza, Alessia Filippone, Laura Cucinotta, Irene Paterniti, Alberto Repici, Anna Paola Capra, Salvatore Cuzzocrea, Emanuela Esposito, Michela Campolo

**Affiliations:** Department of Chemical, Biological, Pharmaceutical and Environmental Sciences, University of Messina, Viale Ferdinando Stagno D’Alcontres, 31, 98166 Messina, Italy

**Keywords:** post-operative pain (PO), DMF, NMDA, calcium

## Abstract

The management of post-operative (PO) pain has generally been shown to be inadequate; therefore, acquiring a novel understanding of PO pain mechanisms would increase the therapeutic options available. There is accumulating evidence to implicate *N*-methyl-d-aspartate (NMDA) receptors in the induction and maintenance of central sensitization during pain states by reinforcing glutamate sensory transmission. It is known that DMF protects from oxidative glutamate toxicity. Therefore, NMDA receptor antagonists have been implicated in peri-operative pain management. Recent advances demonstrated that dimethyl fumarate (DMF), a non-opioid and orally bioavailable drug, is able to resolve neuroinflammation through mechanisms that drive nociceptive hypersensitivity. Therefore, in this study, we evaluated the role of DMF on pain and neuroinflammation in a mouse model of PO pain. An incision of the hind paw was performed, and DMF at two different doses (30 and 100 mg/kg) was administered by oral gavage for five consecutive days. Mechanical allodynia, thermal hyperalgesia and locomotor dysfunction were evaluated daily for five days after surgery. Mice were sacrificed at day 7 following PO pain induction, and hind paw and lumbar spinal cord samples were collected for histological and molecular studies. DMF administration significantly reduced hyperalgesia and allodynia, alleviating motor disfunction. Treatment with DMF significantly reduced histological damage, counteracted mast cell activation and reduced the nuclear factor kappa-light-chain-enhancer of the activated B cell (NF-κB) inflammatory pathway, in addition to downregulating tumor necrosis factor-α (TNF-α), Interleukin-1β (Il-1β) and Il-4 expression. Interestingly, DMF treatment lowered the activation of NMDA receptor subtypes (NR2B and NR1) and the NMDA-receptor-interacting PDZ proteins, including PSD93 and PSD95. Furthermore, DMF interfered with calcium ion release, modulating nociception. Thus, DMF administration modulated PO pain, managing NMDA signaling pathways. The results suggest that DMF positively modulated persistent nociception related to PO pain, through predominantly NMDA-receptor-operated calcium channels.

## 1. Introduction

Post-operative (PO) pain occurs following tissue injury associated with surgery and should resolve during the healing process, normally within 3 months, after which acute pain is considered to be chronic or persistent [1]. The surgery triggers a multitude of responses in the pain matrix as a result of the sensitization of peripheral and central pathways [2]. Recently, the prevalence of intense acute pain increased among patients undergoing surgery [3]. Inadequately controlled acute PO pain is accompanied by increased morbidity, prolonged recovery time, prolonged duration of opioid use and higher health-care costs, factors that contributed to consider PO a health problem [4]. The progress in PO pain analgesia is probably hampered by inadequate efficacy and collateral effects of the available analgesic drugs. Therefore, it is necessary to better investigate molecular and cellular mechanisms underlying PO pain to find therapeutic approaches for its management. Different research showed the importance of *N*-methyl-d-aspartate (NMDA) receptors for the induction and maintenance of central sensitization during pain states; pain associated with peripheral tissue or nerve injury is related to activation of NMDA receptors (NMDARs), and consistent with this, NMDAR antagonists have been exhibited to successfully alleviate pain-related behavior [5,6]. NMDARs have an important role in persistent inflammatory pain by reinforcing glutamate sensory transmission [7]; specifically, glutamate represents the main excitatory neurotransmitter in the nervous system of adult mammals, involved in pain transmission from the periphery to the brain and in central sensitization, which is associated with chronic pain [8]. It is known that glutamate toxicity is counteracted by dimethyl fumarate (DMF), the most pharmacologically effective molecule among the fumaric acid esters (FAEs). DMF is a non-opioid and orally bioavailable drug that alleviates nociceptive hypersensitivity induced by peripheral nerve injury, also resolving neuroinflammation and mitochondrial dysfunction that drive nociceptive hypersensitivity [9]. DMF is currently used as an oral therapeutic agent for the treatment of relapsing forms of multiple sclerosis [10,11] and shows favorable effects in preclinical models of neuroinflammation, neurodegeneration and toxic oxidative stress [12,13,14]. Therefore, we speculated that pharmacological treatment with DMF targeting NMDA receptors may be an effective strategy to counteract PO pain.

## 2. Materials and Methods

### 2.1. Animals

Male adult CD1 mice (20–30 g; Envigo, Udine, Italy) were accommodated in a controlled environment and fed with standard rodent chow and water. Animals were housed in stainless steel cages in a room kept at 22 ± 1 °C with a 12 h light/12 h dark cycle and acclimatized to their environment for 1 week. The study was approved by the University of Messina review board for the care of animals. All animal experiments were carried out in agreement with Italian (DM 116192) and European Union regulations (2010/63/EU amended by Regulation 2019/1010).

### 2.2. Surgical Procedures

Post-operative (PO) pain was performed as explained previously [15]. To induce anesthesia, the animal was put in an isoflurane induction chamber that included an isoflurane dispenser, waiting for approximately 30 s or until the mouse could no longer right itself. Additionally, the animal was anesthetized with 2% inhaled isoflurane by placing the head of the mouse into the nose cone attached to the isoflurane dispenser and covering the mouse with sterile Press’n Seal, except the hind paw selected for operation [16]. Briefly, a 1 cm incision was applied in the plantar hind paw. The underlying muscle was upraised and longitudinally incised, using a 6-0 nylon suture to close the skin. The control group was subjected to anesthesia, but no cut was performed. 

### 2.3. Experimental Groups

Mice were casually divided in the following groups:

Group 1: Sham + vehicle: mice were sedated, without being subjected to cutting. Vehicle solution (saline) was administered daily for 5 days, by oral gavage;
Group 2: Sham + DMF 100 mg/Kg: mice were anesthetized but no incision was performed. DMF (100 mg/Kg) was administered daily for 5 days, by oral gavage;
Group 3: PO pain + vehicle: mice were subjected to surgical procedure and saline was administered daily for 5 days, by oral gavage;
Group 4: PO pain + DMF 10 mg/Kg: mice were subjected to surgical procedure and DMF 10 mg/Kg was administered daily for 5 days, by oral gavage;
Group 5: PO pain + DMF 30 mg/Kg: mice were subjected to surgical procedure and DMF 30 mg/Kg was administered daily for 5 days, by oral gavage;
Group 6: PO pain + DMF 100 mg/Kg: mice were subjected to surgical procedure and DMF 30 mg/Kg was administered daily for 5 days, by oral gavage.

The minimum number of mice for every technique was estimated with the statistical test “ANOVA: Fixed effect, omnibus one-way” with G-power software. This statistical test generated a sample size equal to *n* = 8 mice for each technique and *n* = 24 for each group [17]. Data related to Group 2 (sham + DMF 100 mg/Kg) are not shown because they are similar to the data obtained from control animals, as we have already observed in our previous works on DMF [12,13]. DMF doses and administration route were chosen based on the literature and our studies [12,13,18]. Behavioral analyses were performed daily, and animals were sacrificed 7 days after surgery. Hind paw and lumbar spinal cords were harvested for histological and molecular analyses.

### 2.4. Behavioral Analysis

#### 2.4.1. Mechanical Hyperalgesia

Von Frey filament test was engaged to assess hypersensitivity to a mechanic stimuli, as previously described [19].

#### 2.4.2. Thermal Hyperalgesia

A plantar test was employed to evaluate thermal hyperalgesia, as previously indicated [12].

#### 2.4.3. Motor Coordination

Rotarod test was used to assess motor synchronization [20]. 

### 2.5. Tissue Processing and Histological Analysis

Paw and lumbar spinal cord tissues from the perilesional zone were taken 7 days after surgery and processed for hematoxylin and eosin coloration, as previously described [21,22]. Quantitative assessments of histopathologic inflammation were made and were scored on a 5-point scale. The scores from all sections of each hind paw and lumbar spinal cord were averaged to give a final score for individual mice. Each histological analysis was completed in a blinded fashion. Representative images are shown.

### 2.6. Immunohistochemical Analysis

Hind paw and lumbar spinal cord tissue sections were processed for immunohistochemical analysis, as previously described [23]. Sections were incubated overnight with: IL-1β (sc-12742, 1:100), scaffolding protein postsynaptic density protein-95 (PSD95) (sc-32291, 1:100) and anti-NMDAε2 (NR2B) (sc-365597, 1:100). To verify antibody-binding specificity, control slices were incubated with only primary antibody or secondary antibody, neither of which gave positive staining. The percentage of positive staining was detected using a computerized image analysis system (Leica QWin V3, Cambridge, UK). The images were acquired using an optical microscope (Carl Zeiss Vision Italia S.p.a, Italy, Axio Vision). For graphic display of densitometric analyses, the percentage (%) of positive staining (brown staining) was measured by computer-assisted color image analysis (Leica Qwin V3, UK). The percentage area of immunoreactivity (determined by the number of positive pixels) was expressed as a percentage (%) of the total tissue area (red staining) at 20× magnification. For immunohistochemistry, the images were shown at a magnification of 20× (50 μm of the bar scale) and 40× (20 μm of the bar scale). A systematic uniform random sampling (SURS) method was used to avoid observer-dependent sampling variation and selected a limited number of at least five regions of interest (ROIs) on each whole-slide image.

### 2.7. Toluidine Blue Staining

For evaluation of the number and degranulation of mast cells, paw and lumbar spinal samples were stained with toluidine blue (#05-M23001, Bio-Optica, Milan, Italy), as previously mentioned [24].

### 2.8. Western Blot Analysis

Hind paw and lumbar spinal cord tissues were homogenized, and Western blots were performed as already mentioned [25]. Specific primary antibodies: anti-NFκB (sc-8008, 1:500), anti-IκBα (sc-7271, 1:500), anti-Il-18 (sc-133127, 1:1000), anti-NMDAε2 (NR2B) (sc-365597, 1:500), anti-NMDAζ1 (NR1) (sc-1467, 1:500) and anti-PSD93 (sc-515252, 1:500) were tested. Signals were detected with an enhanced chemiluminescence detection system (Super-SignalWest Pico Chemiluminescent Substrate, Pierce, Monza, Italy). The relative expression of the protein bands was quantified by densitometry with Bio-Rad ChemiDoc XRS software (Bio-Rad, Milan, Italy) and standardized to β-actin or lamin A/C levels. Images of blot signals were imported to analysis software (v2003, Image Quant TL, Amersham Biosciences, Freiburg, Germany). 

### 2.9. ELISA

TNF-α and IL-4 levels were measured in both paw and lumbar spinal cords by enzyme-linked immunosorbent assay (ab208348 and ab100710, respectively, Abcam), according to the manufacturer instructions in pg/mL.

### 2.10. Calcium Quantification

Calcium Assay Kit (ab102505, Abcam) was used to quantify the intracellular calcium content in hind paw and lumbar spinal cord tissues, according to the manufacturer’s instructions.

### 2.11. Statistical Evaluation

All values are expressed as mean ± standard deviation (SD). For in vivo studies, N symbolizes the number of animals subjected to the study. The results were graded using one or two-way analysis of variance followed by a Bonferroni post hoc test for multiple comparisons. A *p*-value of less than 0.05 was considered significant. 

## 3. Results

### 3.1. DMF Modulated Mechanical Allodynia, Thermal Hyperalgesia and Motor Dysfunction Induced by PO Pain

**Figure 1:**
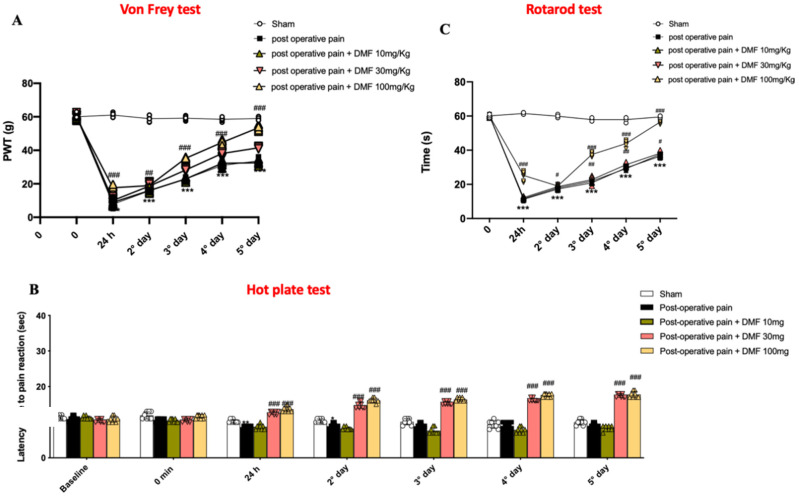
DMF administration reduced mechanical allodynia, thermal hyperalgesiaand motor dysfunction. Von Frey test (**A**), hot plate test (**B**) and rotarod test (**C**) were performed daily from 0 to 5 days. A *p*-value of less than 0.05 was considered significant; *** *p* < 0.001 vs. Sham, ### *p* < 0.001, ## *p* < 0.01 and # *p* < 0.05 vs. PO pain.

Surgical insults, such as hind paw incision, directly activate the peripheral nociceptors, resulting in an increase in central neuronal excitability, leading to allodynia [26]. The hyperalgesia consequential to post-surgery is the result of the hyperexcitability of pain processing at high brain centers, clinically expressed by increased intensity of or abnormal pain sensation [27]. Behavioral studies were executed in order to evaluate the effects of DMF on pain perception. Mechanical allodynia (Figure 1A) and thermal hyperalgesia (Figure 1B) were already strongly augmented 24 h after the surgical incision, gradually decreasing through the five following days. DMF treatment, already after 24 h and significantly for the consecutive five days, increased the response to Von Frey stimulation and to withdrawal latency, augmenting latency time to pain reaction at day four after surgery in the same way for both doses of 30 and 100 mg/kg (Figure 1A,B). A rotarod test was performed to assess motor function (Figure 1C). Impairments in motor coordination were observed in mice 24 h after PO pain induction and took five days to recover. DMF treatment, better at the dose of 100 mg/kg, already notably improved locomotor activity after 24 h and daily until the fifth day (Figure 1C).

### 3.2. Effects of DMF Treatment on Histological Changes Induced by PO Pain

**Figure 2:**
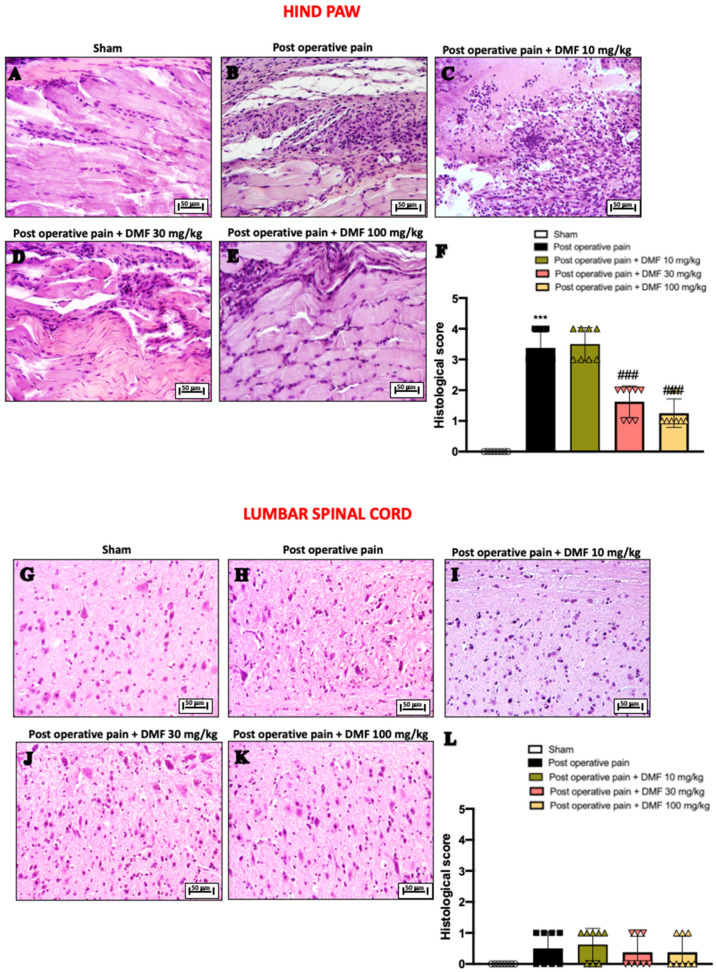
DMF treatment reduced histological damage following PO pain induction. Histological analysis of hind paw: sham (**A**), PO pain (**B**), DMF 10 mg/kg (**C**), DMF 30 mg/kg (**D**,**E**), DMF 100 mg/kg and histological score (**F**). Histological analysis of lumbar spinal cord: sham (**G**), PO pain (**H**), DMF 10 mg/kg (**I**), DMF 30 mg/kg (**J**), DMF 100 mg/kg (**K**) and histological score (**L**). Magnification at 20×. A *p*-value of less than 0.05 was considered significant; *** *p* < 0.001 vs. sham, ### *p* < 0.001 vs. PO pain.

Surgical incision on the hind paw provoked histopathological changes in paw tissue 7 days after the induction, characterized by diffused focal inflammatory cells’ infiltration and interstitial oedema (Figure 2B, see histological score Figure 2F), compared to control mice that preserved the typical architecture of paw tissue with collagen fibers and the presence of fibroblasts (Figure 2A, see histological score Figure 2F). The histopathological severity of the inflammatory response in paw tissue specimens was significantly reduced by the treatment with DMF 30 and 100 mg/kg (Figure 2D,E, see histological score Figure 2F), without obtaining benefit from the treatment at the lower dose of 10 mg/kg (Figure 2C, see histological score Figure 2F). In addition, given that the lumbar spinal cord is a central gateway for peripheral pain signs [28], to better highlight the involvement of the spinal cord in motor functional impairment induced by surgical incision, the histological structure of the lumbar spinal cord was evaluated. No significant morphological changes were observed between the different experimental groups (Figure 2G–K see histological score Figure 2L).

### 3.3. Effects of DMF Treatment on NF-κB Pathway Activation Induced by PO Pain

The pathophysiology of PO pain is unique and involves inflammation and tissue damage [29]; particularly, many data suggest that NF-κB plays a crucial role on inflammatory PO pain [30].

**Figure 3:**
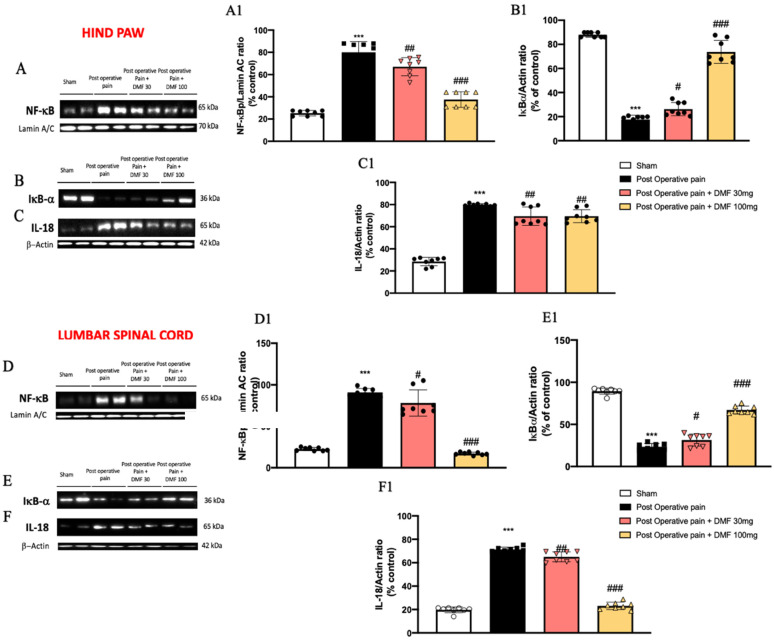
DMF treatment modulated NF-κB pathway. Western blot analysis on hind paw samples of: nuclear NF-κB expression (**A**,**A1**), cytosolic IκB-α (**B**,**B1**) and IL-18 (**C**,**C1**) expression. Western blot analysis on lumbar spinal cord samples of: nuclear NF-κB expression (**D**,**D1**), cytosolic IκB-α (**E**,**E1**) and IL-18 (**F**,**F1**) expression. A *p*-value of less than 0.05 was considered significant; *** *p* < 0.001 vs. sham, ### *p* < 0.001, ## *p* < 0.01 and # *p* < 0.05 vs. PO pain.

Western blot analysis on both hind paw and lumbar spinal cord tissues showed reduced IκB-α expression in the PO pain injured group compared to the control group (Figure 3B,E, see graphs in Figure 3(B1,E1)). DMF treatment significantly increased cytosolic IκB-α expression, restoring it to basal levels when administered at the dose of 100 mg/kg, in both hind paw and lumbar spinal cord tissues (Figure 3B,E, see graphs in Figure 3(B1,E1)). In the nuclear fraction, NF-κB expression resulted to be increased in the damaged group compared to the sham animals (Figure 3A,D, see graphs in Figure 3(A1,E1)). DMF treatment significantly reduced nuclear NF-κB expression 7 days after surgery, better when administered at the higher dose of 100 mg/kg, in both hind paw and lumbar spinal cord tissues (Figure 3A,D, see graphs in Figure 3(A1,E1)). Inflammatory mediators that are part of the NF-κB-mediated pathway influence pain directly or by interaction with other components, such as IL-18 [31]. Interestingly, increased levels of IL-18 were detected in hind paw and lumbar spinal cord tissues from PO pain injured animals, compared to the control group (Figure 3C,F, see graphs in Figure 3(C1,F1)). The tissues levels of IL-18 were strongly reduced by DMF treatment at both doses of 30 and 100 mg/kg (Figure 3C,F, see graphs in Figure 3(C1,F1)).

### 3.4. DMF Modulated Inflammatory Cytokines Released after PO Pain Induction

**Figure 4:**
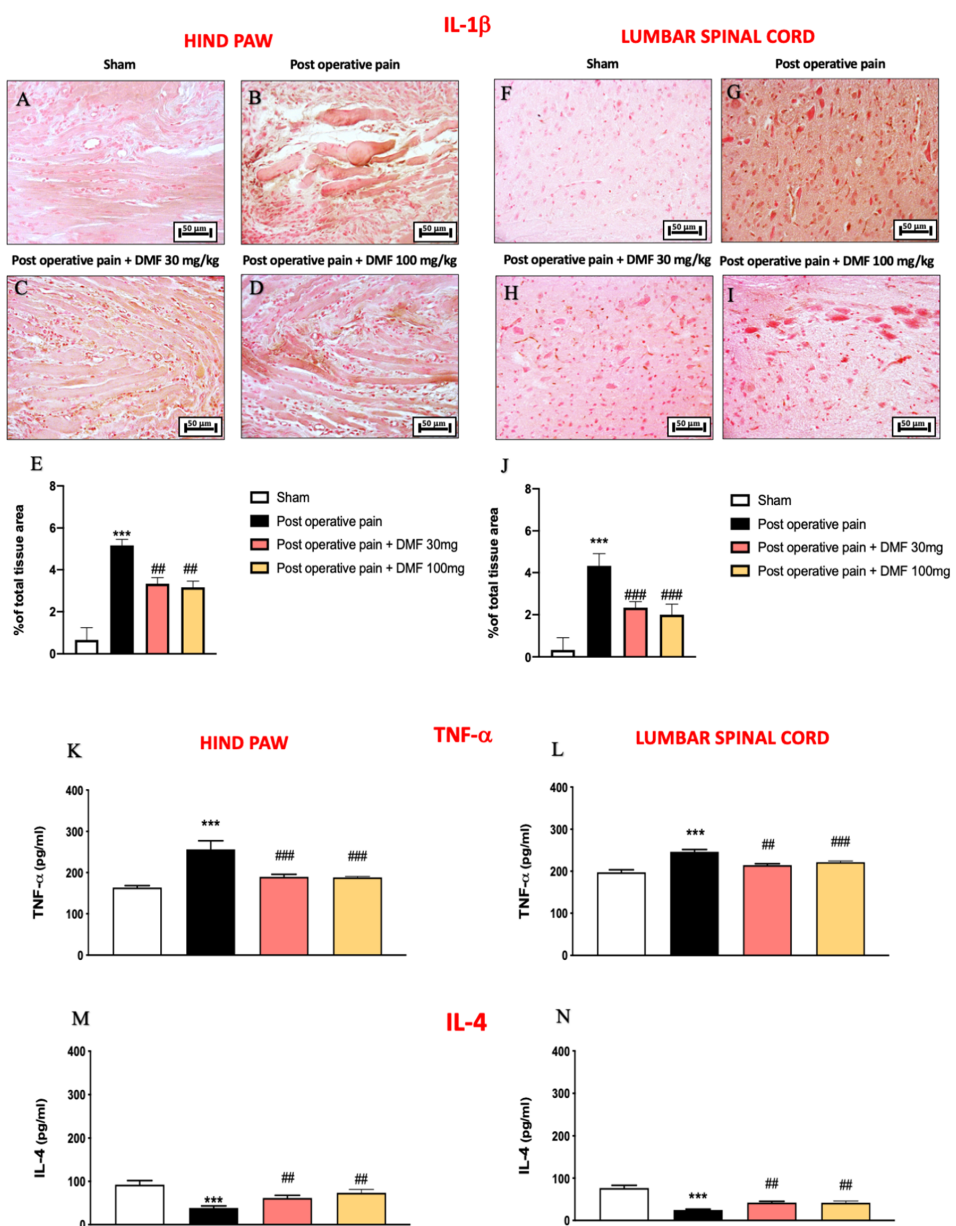
DMF treatment reduced cytokines. Immunohistochemical analysis for IL-1β on hind paw tissue: sham (**A**), PO pain (**B**), DMF 30 mg/kg (**C**), DMF 100 mg/kg (**D**), score (**E**). Immunohistochemical analysis for IL-1β on lumbar spinal cord tissue: sham (**F**), PO pain (**G**), DMF 30 mg/kg (**H**), DMF 100 mg/kg (**I**), score (**J**). Elisa kit for TNF-α (**K**,**L**) and IL-4 (**M**,**N**). A *p*-value of less than 0.05 was considered significant; *** *p* < 0.001 vs. sham, ### *p* < 0.001 and ## *p* < 0.01 vs. PO pain.

Numerous experimental studies provide evidence that pro-inflammatory cytokines, such as IL-1β and TNF-α, induce or facilitate PO pain and hyperalgesia [31]. Evidence demonstrates the importance of IL-1β in both the induction and in the maintenance of pain in chronic states; specifically, IL-1β is a potent mechanical and thermal hyperalgesic agent in many peripheral tissues [32]. In this study, immunohistochemical analysis for IL-1β showed a positive staining in PO pain injured mice, both in hind paw and in lumbar spinal cord tissues (Figure 3G and Figure 4B, see graphs in Figure 4E,J), compared to control (Figure 4A,F, see graphs in Figure 4E,J). DMF administration, in the same way at both doses used, significantly decreased IL-1β positive staining, both in hind paw and lumbar spinal cord tissues (Figure 4C,D,H,I, see graphs in Figure 4E,J). Moreover, TNF-α drives a positive regulatory feedback loop in which TNF-induced inflammation drives further expression of TNF-α, whose role in pathological pain has been extensively reviewed [33,34]. The ELISA kit performed on both hind paw and lumbar spinal cord tissues highlighted a strong increase in the TNF-α quantity in the vehicle group compared to control mice (Figure 4K,L); treatment with DMF, in the same way at both doses of 30 and 100 mg/kg, notably reduced TNF-α amount (Figure 4K,L). The effects of TNF-α could be modulated by IL-4 [35]. Despite some evidence regarding the role of IL-4 as an attractive candidate for treatment of pathological pain due to its broad spectrum of anti-inflammatory actions [36,37], it can serve as a facilitator of pro-inflammatory cytokine expression [38,39]. Interestingly, in this study, an increasing amount of IL-4 was observed in samples from mice subjected to PO pain compared to the control group (Figure 4M,N), while DMF treatment at 30 and 100 mg/kg significantly reduced the quantity of IL-4 (Figure 4M,N).

### 3.5. DMF Role on Mast Cells Released Following PO Pain Induction

**Figure 5:**
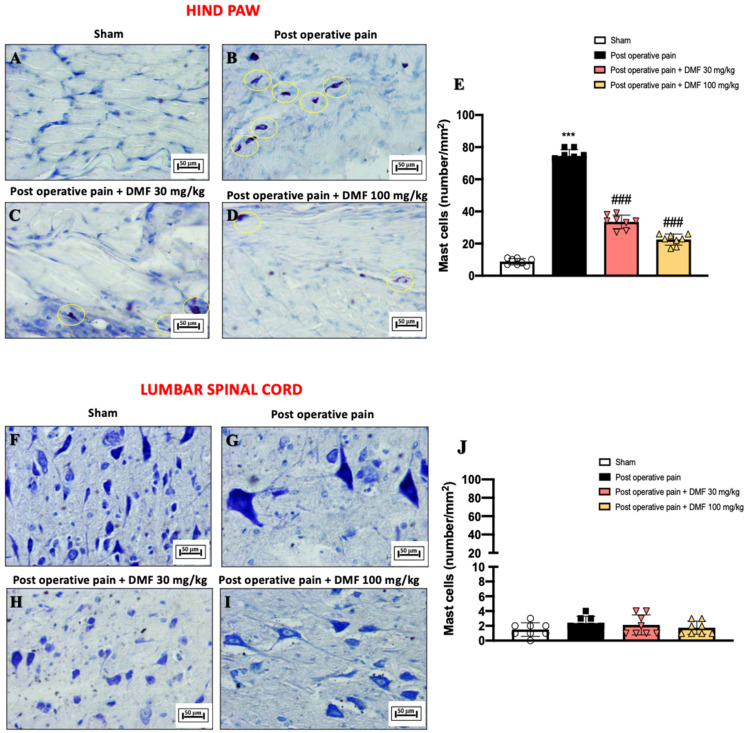
DMF treatment reduced mast cell degranulation following PO pain induction. Toluidine blue staining on hind paw tissue: sham (**A**), PO pain (**B**), DMF 30 mg/kg (**C**), DMF 100 mg/kg (**D**) and score (**E**). Toluidine blue staining on lumbar spinal cord tissue: sham (**F**), PO pain (**G**), DMF 30 mg/kg (**H**), DMF 100 mg/kg (**I**) and score (**J**). Magnification 20×. A *p*-value of less than 0.05 was considered significant; *** *p* < 0.001 vs. sham, ### *p* < 0.001 vs. PO pain.

During inflammatory pain, mast cells are one of the immune cell types involved in the process, playing a key role in the development of hyperalgesia [40]. As shown in Figure 5B and the relative graph in Figure 5E, 7 days after the surgical incision of the hind paw there was a significant increase in mast cell number, compared with the non-injured hind paw of control group (Figure 5A, see graph in Figure 5E). Treatment with DMF at both 30 and 100 mg/kg doses significantly reduced mast cell infiltration compared to the PO pain group (Figure 5C,D, see graph in Figure 5E). Regarding the evaluation of mast cell intrusion in the lumbar spinal cord, no substantial difference was observed between the various experimental groups (Figure 5F–J).

### 3.6. DMF Modulation on NMDA Pathway Activated Following PO Pain Induction

**Figure 6:**
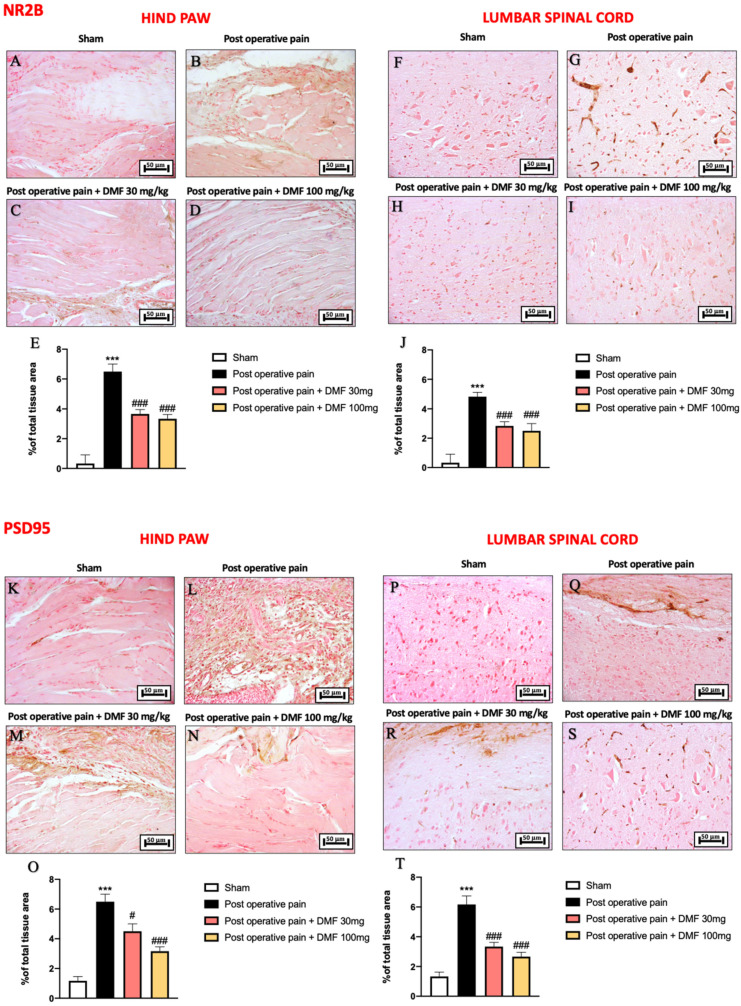
DMF treatment modulated NMDA pathway. Immunohistochemical analysis of NR2B on hind paw tissue: sham (**A**), PO pain (**B**), DMF 30 mg/kg (**C**), DMF 100 mg/kg (**D**), score (**E**). Immunohistochemical analysis of NR2B lumbar spinal cord tissue: sham (**F**), PO pain (**G**), DMF 30 mg/kg (**H**), DMF 100 mg/kg (**I**), score (**J**). Immunohistochemical analysis of PSD95 on hind paw tissue: sham (**K**), PO pain (**L**), DMF 30 mg/kg (**M**), DMF 100 mg/kg (**N**), score (**O**). Immunohistochemical analysis of PSD95 on lumbar spinal cord tissue: sham (**P**), PO pain (**Q**), DMF 30 mg/kg (**R**), DMF 100 mg/kg (**S**), score (**T**). Magnification 20×. A *p*-value of less than 0.05 was considered significant; *** *p* < 0.001 vs. sham, ### *p* < 0.001 and # *p* < 0.05 vs. PO pain.

NMDA receptors (NMDARs) are thought to play an important role in the processes of central sensitization and pathogenesis of neuropathic pain [41]; functional NMDARs are heteromeric complexes mainly consisting of NR1 and NR2 (NR2A-D) subunits. PSD95 enhances NMDAR clustering at synapses and inhibits NR2B-mediated internalization. Binding of NMDA receptor subunits by PSD95 can facilitate downstream intracellular signaling and modulate receptor stability, promoting chronic pain [42]. Low positive staining for NR2B was identified in the hind paw and lumbar spinal cord from control mice (Figure 6A,F, see graphs in Figure 6E,J). However, 7 days following hind paw incision, increased NR2B immunoreactivity was detected in the PO pain group (Figure 6B,G, see graphs in Figure 6E,J). DMF treatment at both doses of 30 and 100 mg/kg was able to significantly reduce positive staining for NR2B in the hind paw and lumbar spinal cord (Figure 6C,D,H,I, see graphs in Figure 6E,J).

Interestingly, an increase in PSD95 positive expression was found in the PO pain subjected group (Figure 6L,Q, see graphs in Figure 6O,T), compared to the control (Figure 6K,P, see graphs in Figure 6O,T). A significant reduction in PSD95 immunoreactivity in hind paw tissue was observed following DMF treatment, better when administered at the dose of 100 mg/kg (Figure 6N, see graph in Figure 6O) than 30 mg/kg (Figure 6M, see graph in Figure 6O). Similar effects at different doses of DMF (30 and 100 mg/kg) were observed in lumbar spinal cord tissues (Figure 6R,S, see graph in Figure 6T). 

### 3.7. DMF Modulation on NMDA-Receptor-Operated Calcium Channels in PO Pain

**Figure 7:**
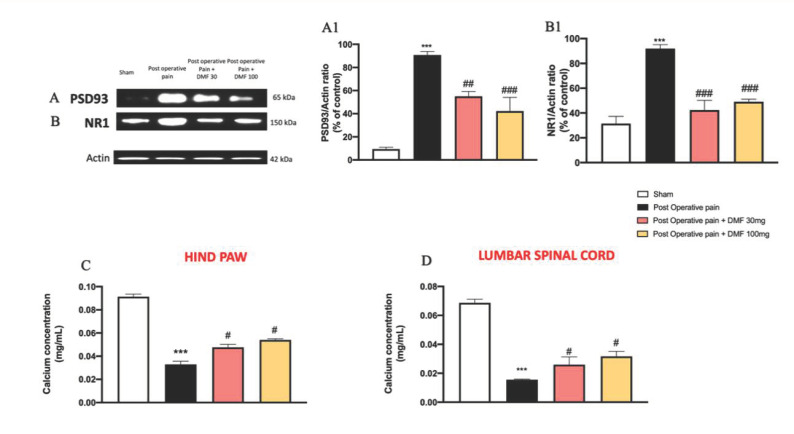
DMF treatment modulated NMDA-receptor-operated calcium channels. Western blot analysis on hind paw samples of cytosolic PSD93 (**A**,**A1**) and NR1 expression (**B**,**B1**). ELISA kit for calcium concentration on hind paw tissue (**C**) and lumbar spinal cord tissue (**D**). A *p*-value of less than 0.05 was considered significant; *** *p* < 0.001 vs. sham, ### *p* < 0.001, ## *p* < 0.01, # *p* < 0.05 vs. PO pain.

The different molecular mechanisms of NMDA receptors depend on the association of the obligatory NR1 subunit with the different NR2 subunits; generally, there are two major subtypes: NR1/NR2A and NR1/NR2B [43]. In this context, it has been accepted that PSD93 takes effect mainly through modulating NMDA receptors and their downstream signals [44]; particularly, PSD93 was critical for NMDAR-mediated postsynaptic function and NMDAR-dependent persistent pain [45]. Western blot analysis showed a significant increase in PSD93 and NR1 protein levels in lumbar spinal cord tissues from the PO pain group compared to control (Figure 7A,B, see graphs in Figure 7(A1,B1)); DMF treatment at both doses of 30 and 100 mg/kg significantly reduced PSD93 and NR1 expression levels (Figure 7A,B, see graphs in Figure 7(A1,B1)).

Furthermore, NMDAR activation results within milliseconds in the opening of the integral ion channel that has a high permeability for calcium ions (Ca^++^), neuronal depolarization and subsequent activation of NMDA receptor downstream signaling pathways [46]. NMDA-receptor-induced Ca^++^ influx has also been shown to promote rapid inactivation of NMDARs, as a negative feedback control system to regulate Ca^++^ influx [47]. The Ca^++^ concentration resulted to be significantly lowered in tissues from the PO pain group compared to control (Figure 7C,D) in both hind paw and lumbar spinal cord tissues. DMF treatment at both doses of 30 and 100 mg/kg reduced Ca^++^ concentration (Figure 7C,D).

## 4. Discussion

PO pain is considered one of the most deceitful forms of surgical pain. The lack of patient care in the post-surgical phase results in poorly controlled post-operative pain. Inadequate PO pain management provokes harmful effects on quality of life, contributing to the development of chronic post-surgical pain [48]. Despite the knowledge regarding the molecular biology and neurology of PO pain, the clinical management of PO pain seems to be unsuccessful [49]. The improvement in therapeutical approach for PO pain analgesia is probably disadvantaged by limited efficiency of the accessible analgesic drugs and their related side effects [50]. Therefore, investigating the molecular and cellular mechanisms at the base of PO pain is necessary. FAEs represent a valid approach against fibromyalgia and other pathologies associated with pain or caused by inflammatory processes [51]; DMF, the most pharmacologically effective molecule among the FAEs, alleviated nociceptive hypersensitivity induced by peripheral nerve injury, also resolving neuroinflammation and nociceptive hypersensitivity. NMDARs have an important role in persistent inflammatory pain by reinforcing glutamate sensory transmission [7,52], and it is known that DMF protects from oxidative glutamate toxicity [53]. Therefore, the aim of this study was to investigate the pharmacological role of DMF, through NMDA receptors, to counteract the hypersensitivity and inflammatory state in an in vivo mouse model of PO pain.

Severe acute PO pain is a risk factor for chronic post-surgical pain, raising more awareness regarding the importance of adequate peri-operative pain management [54]; generally, mechanical hyperalgesia occurs at the side of the incision and in an area surrounding the injury [55], and heat hyperalgesia results to be prominent at the site of the incision. The anti-nociceptive effects of DMF were demonstrated in this study through an increase in latency time to pain reaction in the same way at both doses of 30 and 100 mg/kg. Moreover, functional instability with motor coordination impairments of the core musculature is the clinical assumption after an episode of pain related to surgery [56]; oral DMF treatment notably improved locomotor activity, restoring motor impairments. A surgical incision on the hind paw to induce PO pain provoked histopathological changes in paw tissue seven days after the induction; the histopathological severity of the inflammatory response in paw tissue specimens was significantly reduced by the treatment with DMF, while no significant morphological changes were observed in lumbar spinal cord tissue. 

Cytokines are key modulators of inflammatory responses and show an important function in the defense and repair mechanisms following trauma; specifically, after surgical injury, an immuno-inflammatory response is initiated immediately, and cytokines rapidly appear and function as a regulator of immunity, playing important roles in post-operative organ dysfunction and pain modulation [57]. In this study, we highlighted the capacity of DMF treatment to significantly decrease IL-1β positive staining and reduce TNF-α, IL-4 and IL-18 expression levels, both in hind paw and lumbar spinal cord tissues. Cytokines are produced by immune cells, including mast cells [58], and it is known that these last represent important first responders in protective pain responses that provoke withdrawal from intense, noxious environmental stimuli [59]; treatment with DMF significantly reduced mast cell infiltration. The notable capacity of DMF to counteract post-surgery inflammation was also demonstrated through the protective modulation of the NF-κB pathway. Glutamate-gated ionotropic NMDARs and voltage-gated Ca^++^ channels are important routes to mediate NF-κB activation during an inflammatory state [60], and NMDARs represent an essential step in initiating and maintaining the central sensitization and pain hypersensitivity after tissue injury [61].

NMDARs consist of heterotetrameric assemblies of different subunits within a repertoire of three subtypes, whereas NR1 distributes ubiquitously in the central nervous system (CNS), while NR2 subunits exhibit regional distribution and the amount of expression is developmentally related. In particular, NR2B-containing NMDA receptors function as promising targets for treatment of algesia [62]. In this study, we demonstrated that DMF treatment significantly reduced positive staining for NR2B and NR1 expression levels in both hind paw and lumbar spinal cord tissues. The membrane-associated guanylate kinases (MAGUKs) PSD95 and PSD93 are thought to have crucial roles in receptor trafficking and formation of the NMDA receptor-associated signaling complexes involved in synaptic plasticity [63]. PSD95 enhances NMDAR clustering at synapses and inhibits NR2B-mediated internalization; co-expression of PSD95 with NMDARs increases surface expression of NMDARs and enhances synaptic NMDAR function [45]. In this research, treatment with DMF considerably reduces PSD95 and PSD93 expression levels, suggesting a positive reduction in pain relief. Furthermore, activation of NMDARs results within milliseconds in the opening of the integral ion channel that has a high permeability for calcium ions (Ca^++^), neuronal depolarization and subsequent activation of NMDA receptor downstream signaling pathways [46]. NMDA receptor-induced Ca^++^ influx has also been shown to promote rapid inactivation of NMDARs as a negative feedback control system to regulate Ca^++^ influx [47]. DMF treatment at both doses of 30 and 100 mg/kg increased Ca^++^ concentration in both hind paw and lumbar spinal cord tissues, highlighting that the pleiotropic changes in cellular calcium homeostasis caused by DMF treatment likely contribute to its analgesic effects.

## 5. Conclusions

In conclusion, these results highlighted that DMF positively modulated persistent nociception related to PO pain through predominantly NMDA-receptor-operated calcium channels, carrying out an action both centrally and peripherally, suggesting DMF as a valid alternative approach to remodulate PO pain.

## Data Availability

The data presented in this study are available on request from the corresponding author.
